# Supplementary material for: Identification and functional analysis of non-coding regulatory small RNA FenSr3 in Bacillus amyloliquefaciens LPB-18
Source: PeerJ. 2023 May 15;11:e15236. doi: 10.7717/peerj.15236 (PMC10194069; doi:10.7717/peerj.15236)
Supplement: Supplemental Information 4 [file peerj-11-15236-s004.zip › KO/CK-vs-T1_map/map00902.html]

KEGG PATHWAY: Monoterpenoid biosynthesis - Reference pathway


|  |  |
| --- | --- |
| **Monoterpenoid biosynthesis - Reference pathway** |  |

[
Pathway menu
| Organism menu
| Pathway entry
| Show description
| User data mapping
]

Monoterpenoids (C10 terpenoids) are a group of terpenoids consisting of two isoprene units. They are derived from geranyl diphosphate (GPP). Most monoterpenoids are volatile oils with highly distinctive aromas and flavors, such as essential oils, turpentine, and oleoresins of coniferous plants. This map shows some examples. The monoterpene ketone l-menthone is specifically converted to l-menthol and d-neomenthol in mature peppermint leaves. The iridoids constitute a family of highly oxygenated monoterpenes, mixtures of which are present in many medicinal plants, such as valerian. They are derived from geraniol or nerol via oxidation of a terminal methyl group. The cyclopentane ring of loganin can itself be cleaved in a further P450-dependent step, leading to secologanin, which provides the carbon skeleton for many powerfully bioactive secondary metabolites of indole alkaloids.

|  |  |  |
| --- | --- | --- |
| Reference pathway | 184% 150% 122% 100% 82% 67% 55% | 图片下载 |
